# Combined Effects of Superabsorbent Polymers, Biochar and Humic Acid on Soil Water Salt Dynamics and *Melilotus officinalis* Growth

**DOI:** 10.3390/plants15101514

**Published:** 2026-05-15

**Authors:** Yongle Tu, Kexin Guo, Shuying Zhao, Yongping Cheng, Ying Liu, Jiaqiang Cao, Xiaojiao Wang, Xinhui Han, Chengjie Ren, Yongzhong Feng, Gaihe Yang

**Affiliations:** 1 College of Agronomy, Northwest A&F University, Yangling 712100, China; 2023050118@nwafu.edu.cn (Y.T.); 2023050134@nwafu.edu.cn (K.G.); 2023055012@nwafu.edu.cn (S.Z.); 2023055127@nwafu.edu.cn (Y.C.); 2023055019@nwafu.edu.cn (Y.L.); 2023055005@nwafu.edu.cn (J.C.); hanxinhui@nwsuaf.edu.cn (X.H.); rencj1991@nwsuaf.edu.cn (C.R.); fengyz@nwsuaf.edu.cn (Y.F.); ygh@nwsuaf.edu.cn (G.Y.); 2 Shaanxi Engineering and Technological Research Center of Cyclic Agriculture, Yangling 712100, China

**Keywords:** superabsorbent polymer, biochar, humic acid, *Melilotus officinalis* (L.) Lam., saline–alkali soil, water–salt transport, amelioration effects

## Abstract

Soil salinization is one of the most severe forms of land degradation in arid and semi-arid regions, posing substantial threats to agroecosystem stability and food security. In this study, saline–alkali soil collected from the Wuding River Basin in Yulin, Shaanxi Province was used to construct a three-factor amendment system comprising superabsorbent polymers (SAP), biochar, and humic acid. A systematic assessment was conducted to elucidate their combined effects on soil water–salt transport and crop growth. Results from one-dimensional constant-head infiltration experiments using indoor soil columns demonstrated that the application of amendments significantly increased cumulative infiltration and improved the uniformity of wetting-front advancement. Specifically, the treatments regulated the redistribution of salts within the soil profile; while surface salinity reduction varied, the leaching efficiency was significantly enhanced in the A2B2C2 treatment. Soil bulk density (BD) showed dynamic fluctuations during the growth cycle, peaking at 1.628 cm^−3^ during the branching stage, while high-rate biochar (A3) reduced BD by up to 13.64% compared to the control by the initial flowering stage. Fitting results based on the Philip and Kostiakov models further indicated that the combined amendment strategy—particularly the A2B2C2 treatment (30 kg/ha SAP, 15,000 kg/ha biochar, and 600 kg/ha humic acid)—markedly enhanced both the initial infiltration rate and the steady infiltration capacity. Field experiments corroborated the indoor findings: plant height and dry biomass of *Melilotus officinalis* (L.)Lam. were significantly higher under amendment treatments than in the control, driven by improved water availability, mitigated salt stress, and enhanced soil structure. Single-factor and multi-factor interaction analyses revealed that SAP exerted pronounced effects during early growth stages, whereas biochar and humic acid contributed more substantially during the middle to late stages through sustained regulatory functions. Collectively, the results demonstrate that the combined application of SAP, biochar, and humic acid improves the water–salt regime of saline–alkali soils through a coupled “water–salt–structure–plant” mechanism, ultimately enhancing crop productivity. This study provides both theoretical insights and practical guidance for the amelioration of saline–alkali soils.

## 1. Introduction

Soil salinization is one of the most severe forms of land degradation in arid and semi-arid regions worldwide, with more than 1 billion hectares of land currently affected by salinity-related degradation [1,2]. High concentrations of soluble salts and the resulting elevated osmotic potential not only inhibit soil water infiltration and retention capacity but also disrupt soil aggregate structure [3]. The excessive accumulation of Na^+^ and Cl^−^ ions exerts severe physiological stress on crops, disrupting ionic balance and inhibiting gas exchange, which ultimately leads to stunted growth and reduced survival rates [4]. This results in yield losses exceeding 40% in some regions, posing a substantial threat to agroecosystem stability and global food security [5,6]. Water–salt transport is a core mechanism governing the formation and evolution of saline–alkali soils, jointly regulated by soil physicochemical properties, water dynamics, ionic composition, and amelioration practices [7]. Therefore, the development of materials capable of enhancing soil water retention, promoting downward salt leaching, and suppressing salt return is of significant importance for improving the productivity of saline–alkali lands [8,9].

Soil water migration is influenced by a combination of factors, including soil texture, structural stability, and organic matter content [10]. In saline–alkali soils, the generally high pH facilitates the replacement of divalent cations such as Ca^2+^ and Mg^2+^ by exchangeable Na^+^ on colloidal surfaces [11]. The displaced divalent cations readily precipitate under strongly alkaline conditions, reducing the soil’s effective pore volume [12]. Meanwhile, exchangeable Na^+^ substantially enhances hydration and dispersion of soil colloids, leading to aggregate breakdown and a shift from macropores to finer pores as the dominant pore type [13,14]. This degradation of pore structure increases hydraulic resistance, decreases water transmission under a given hydraulic gradient, and ultimately results in lower hydraulic conductivity in saline–alkali soils than in non-saline soils [15]. Consequently, saline–alkali soils exhibit reduced cumulative infiltration, lower infiltration rates, and more rapid infiltration decay [16,17]. These observations clearly indicate that soil salinity and pH exert critical influences on infiltration mechanisms and parameters, making them key determinants of hydraulic behavior in saline–alkali soils [18].

In recent years, superabsorbent polymer (SAP), biochar, and humic acid have emerged as prominent soil amendments for improving soil structure, regulating water–salt transport, and enhancing crop growth in saline–alkali environments [19,20]. SAP, as a class of highly absorbent polymers, can significantly increase soil water-holding capacity, reduce surface evaporation and salt accumulation, and modify pore size distribution and aggregate structure to enhance infiltration capacity [21], thereby altering vertical water–salt migration pathways. Biochar, characterized by its highly porous structure and large specific surface area, improves soil aeration and cation exchange capacity, enhances water retention, and adsorbs ions such as Na^+^ to alleviate salt stress [22,23], while also providing a favorable habitat for microbial activity. Humic acid, a natural macromolecular organic substance rich in reactive functional groups, promotes soil aggregate formation, improves aeration and water retention, and regulates ionic balance through complexation and ion exchange, thereby reducing pH and ameliorating both alkalinity and soluble salt content in saline–alkali soils [24]. However, existing research demonstrates that each amendment individually contributes to improving water–salt transport; their effectiveness is often limited when applied alone under extreme salinity due to the single-action mechanism [25]. For instance, high ionic strength can cause SAP to shrink, and biochar alone may not sufficiently lower the soil pH in highly alkaline conditions [26]. Moreover, high application rates of biochar may increase the risk of CH_4_ (methane) emissions [27]. Combining SAP, biochar, and humic acid is expected to generate synergistic effects: biochar provides a stable structural framework, SAP maintains a dynamic water reservoir, and humic acid facilitates chemical ion exchange. This “physical-chemical” coupled strategy aims to address the multidimensional constraints of saline soils—water scarcity, salt toxicity, and structural collapse—which remains a critical research gap in current literature [28].

*Melilotus officinalis* was selected as the test plant in this study due to its status as a premier pioneer forage for reclaiming saline–alkali land, characterized by high salt tolerance and nitrogen-fixing capacity [29]. Despite its robustness, severe salinity stress still hampers its biomass accumulation by inducing oxidative stress and reducing water use efficiency [30]. Evaluating the growth response of M. officinalis provides a vital biological indicator for the efficacy of the amendment system.

To address these issues, this study establishes a clear research framework based on the hypothesis that the ternary combination of SAP, biochar, and humic acid creates a superior “water–salt–structure” balance. This study used saline–alkali soil from the Wuding River region of Yulin, Shaanxi Province. The specific objectives were: (1) to quantify the microscopic effects of combined amendments on infiltration dynamics and salt redistribution through indoor one-dimensional soil column experiments (fitted by Philip and Kostiakov models); and (2) to validate the effectiveness of these treatments on soil reclamation and *M. officinalis* growth under natural field conditions. Through integrated analyses, this study identifies optimal combinations and elucidates their mechanisms, providing technical guidance for the sustainable amelioration of saline–alkali soils.

## 2. Results

### 2.1. Laboratory Soil Column Experiment

Based on the results in Table 1 a range analysis was conducted to evaluate the effects of different soil amendments on cumulative infiltration and the wetting front advancement distance. In the table, Ki (i = 1, 2, 3) represents the sum of all evaluation index values corresponding to the i-th level of a given factor, where i denotes the number of levels of that factor. The term ki refers to the mean value for each level, calculated as ki = Ki divided by 9. The range R is defined as the difference between the maximum and minimum ki values for a given factor. A larger R value indicates a more significant influence of that factor on the evaluation indices.

#### 2.1.1. Effects of Soil Amendments on Cumulative Infiltration

Range analysis of the cumulative infiltration for each treatment, as shown in Table 2, indicates that the order of influence among the factors is superabsorbent polymer greater than biochar greater than humic acid. Combined with the orthogonal experiment results, cumulative infiltration increased over time in all treatment groups and showed a positive correlation with the application rates of the amendments. When the application rate of the superabsorbent polymer was 60 kg/ha, the application rate of biochar was 30,000 kg/ha, and the application rate of humic acid was 1200 kg/ha, corresponding to treatment T9, the cumulative infiltration of the soil reached its maximum. This suggests that at the observed scales, the synergistic effect of these materials—particularly the high water-retention capacity of SAP and the porosity-improving nature of biochar—effectively expanded the soil’s water-storage matrix.

#### 2.1.2. Effects of Soil Amendments on Wetting Front Advancement

**Figure 1:**
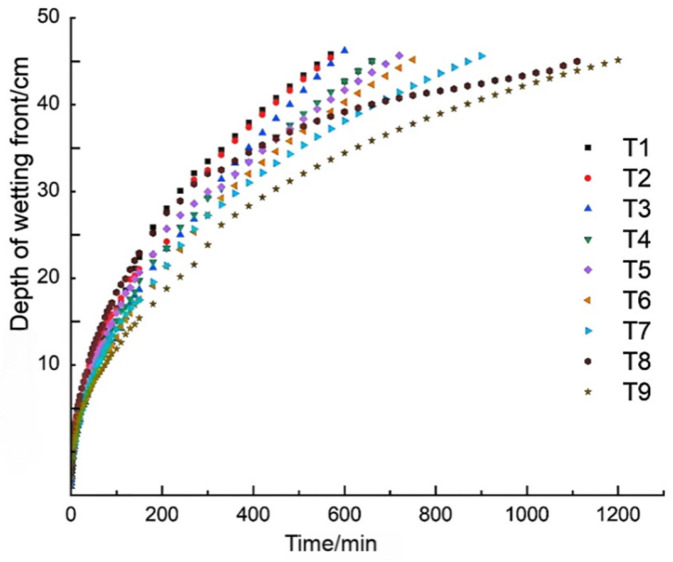
Effect of amendment application on wet front migration.

As illustrated in Figure 1, the variation in soil infiltration rates remained consistent across all treatments, following a typical decline from an initial high rate to a steady state. In the first 30 min, the dry soil condition and high matric potential gradient promoted rapid water movement. However, the application of SAP introduced a dual effect: while its high water-absorption capacity increased soil water potential locally, its swelling volume physically reduced soil porosity. This mechanism, particularly evident in high-rate treatments (e.g., T9), explains the gradual stabilization of infiltration rates as the amendment layer became saturated.

Within the first 100 min of infiltration, the wetting front advancement curves of treatments T1, T3, and T8 showed similar patterns with relatively fast advancement. The curves of treatments T2, T4, T5, and T7 were also similar to each other. In contrast, the wetting front advancement rates of T6 and T9 were noticeably lower than those of the other treatments. After entering the steady infiltration stage, T1 still exhibited the highest wetting front advancement rate, indicating that the application of soil amendments significantly enhanced the initial infiltration efficiency. This also suggests that the superabsorbent polymer primarily improves early infiltration by increasing soil water-holding capacity and modifying pore structure, which facilitates rapid downward movement of water while reducing energy loss during infiltration. These effects create favorable conditions for the subsequent stable advancement of the wetting front. The experimental results demonstrate that the amendments not only increase the initial infiltration rate but also regulate pore structure and water retention, resulting in a more uniform and stable wetting front movement.

As shown in Table 3, during the early stage of infiltration at 100 min, the R values of the wetting front for the different factors followed the order RA greater than RB greater than RC. This indicates that the relative importance of the three factors affecting wetting front advancement distance was superabsorbent polymer greater than biochar greater than humic acid. Under single-factor consideration, the combination A2B1C1, corresponding to 30 kg/ha of superabsorbent polymer, 0 kg/ha of biochar, and 0 kg/ha of humic acid, resulted in the fastest wetting front advancement during the first 100 min.

As infiltration time progressed into the middle stage at 300 min, the R values again followed the order RA greater than RB greater than RC. This confirms that the same ranking of factor importance applied during the mid stage of infiltration. Under single-factor conditions, the optimal combination was A2B2C2, corresponding to 30 kg/ha of superabsorbent polymer, 15,000 kg/ha of biochar, and 600 kg/ha of humic acid, which produced the greatest enhancement in wetting front advancement speed.

#### 2.1.3. Effects of Soil Amendments on Soil Salinity and Moisture Distribution

**Figure 2:**
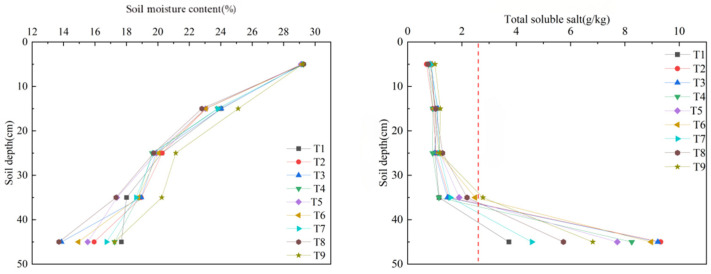
Vertical distribution of soil water content and soil salinity content under different treatments. Note: The red dashed line indicates the initial salt content.

Soil water and salt distribution is an important indicator for evaluating the effectiveness of soil amendments and characterizing soil physical and chemical properties. According to Figure 2, soil moisture content generally decreased with increasing soil depth across all treatments, and the differences among treatments gradually widened. Among all treatments, T9 consistently showed significantly higher soil moisture content at all depths, indicating that the application of soil amendments effectively increased soil moisture and water-holding capacity and improved the uniformity of soil moisture distribution.

Figure 2 also shows that soil salt content increased slowly with depth within the 0 to 40 cm layer, but increased sharply below 40 cm. This pattern was mainly caused by the downward movement of salts with infiltrating water during the infiltration process, followed by salt accumulation in deeper soil layers after infiltration ceased. Compared with the control, the treatments with amendments exhibited higher initial infiltration rates, which enhanced solute transport and resulted in significantly lower salt content in the 0 to 20 cm layer. This indicates that the amendments effectively suppressed salt accumulation in the surface soil during the early stage.

As infiltration time increased and infiltration rate declined, soil salt content in the 30 to 50 cm layer rose rapidly, showing a sudden increase. This suggests substantial salt accumulation in the middle and deeper soil layers. This dynamic process reflects the coupled movement of water and salt, in which downward water movement drives shallow-layer salts toward deeper layers, thereby altering the overall soil water and salt distribution pattern.

In addition, soil salt content increased with higher amendment application rates across all soil layers. In treatment T5, soil salt content increased from 2.14 g per kg at 30 cm to 7.96 g per kg at 50 cm. In treatment T2, the increase was even greater, rising from 1.82 g per kg to 8.93 g per kg, with the highest salt content observed in the 30 to 50 cm layer. These results indicate that appropriate application of soil amendments not only promotes water infiltration but also facilitates the downward movement of salts from shallow layers to deeper layers. This helps alleviate salt stress in the surface soil, improves soil water and salt dynamics, and creates a more favorable water and salt environment for subsequent crop growth.

#### 2.1.4. Simulation of the Infiltration Process and Infiltration Characteristic Parameters

Water infiltration parameters are important indicators for characterizing soil water movement and infiltration behavior. In this study, the sorptivity S in the Philip infiltration model and the integrated shape factor in the one dimensional algebraic model were systematically analyzed. According to the Philip model, the relationship between cumulative infiltration and time can be substituted into the model equation to calculate the sorptivity S, which reflects the initial infiltration rate and the soil water penetration capacity. In the one dimensional algebraic model, the integrated shape factor is obtained by substituting cumulative infiltration, wetting front depth, saturated volumetric water content, and residual volumetric water content into the corresponding calculation equations. This factor characterizes the development of the wetting front and the pattern of soil water distribution, providing a quantitative basis for evaluating the effects of different amendment treatments on soil infiltration performance, as shown in Table 4.

Both infiltration models showed high fitting accuracy when simulating the variation in infiltration rate over time under different amendment conditions, with coefficients of determination *R*^2^ exceeding 0.98. This indicates strong model performance. However, during parameter inversion of the Philip model, the stable infiltration rate A appeared as a negative value. Physically, A represents the soil’s hydraulic conductivity at saturation and cannot be negative. This discrepancy suggests that the standard Philip model may not fully account for the complex matrix suction and swelling pressure exerted by SAP in this specific soil amendment system. Consequently, the Kostiakov model provided a more robust and physically consistent description of the infiltration decay pattern. Its parameter α effectively reflected the degree of infiltration rate decline over time. A smaller α value indicates a slower reduction in infiltration rate, suggesting stronger infiltration persistence. Therefore, in terms of overall fitting performance, the Kostiakov model demonstrated better stability and predictive capability than the Philip model in this study.

In the inverted parameters of the Philip model, all amendment treatments increased the stable infiltration rate compared with the control, indicating that the three amendments, including the superabsorbent polymer, biochar, and humic acid, enhanced the infiltration performance of saline alkali soil and reduced the risk of surface salt accumulation. Regarding sorptivity S, the combination with 0 kg/ha superabsorbent polymer, 15,000 kg/ha of biochar, and 1200 kg/ha of humic acid, corresponding to treatment T2, produced the highest sorptivity. Sorptivity is an important parameter for evaluating the initial water infiltration capacity, and a larger value indicates stronger soil water absorption and a higher initial infiltration rate. Thus, an appropriate combination of amendments can significantly improve water transmission efficiency during the early stage of infiltration.

Further comparison of the treatments showed that the A2B2C2 combination (30 kg/ha SAP, 15,000 kg/ha biochar, and 600 kg/ha humic acid) achieved the best fitting performance in both models. This indicates that this combination had the most pronounced effect on improving water and salt transport in saline alkali soil and represents the optimal amendment scheme under the conditions of this experiment.

### 2.2. Field Experiment

#### 2.2.1. Effects of Soil Amendments on Soil Moisture Content

**Figure 3:**
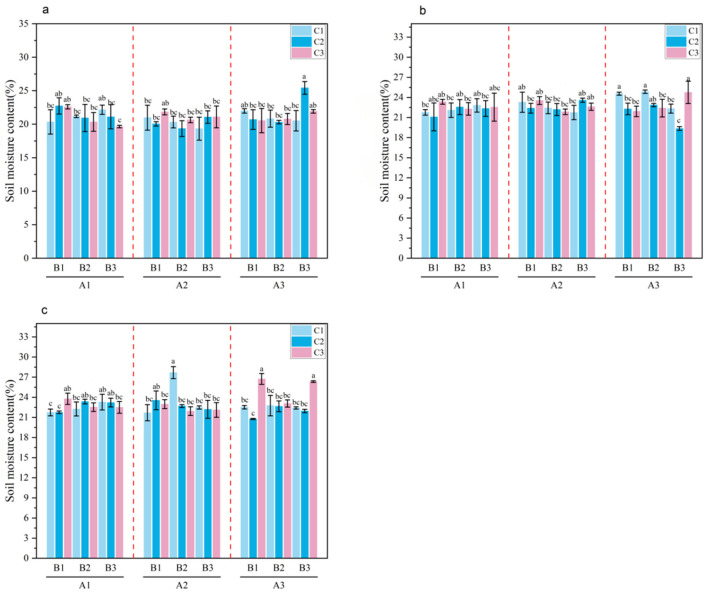
Soil Moisture Content (0–20 cm) during Key Growth Stages of *Melilotus officinalis.* Note: Lowercase letters indicate significant differences among treatments (*p* < 0.05). Panels (**a**–**c**) represent the seedling stage, branching stage, and initial flowering stage of *Melilotus officinalis*, respectively.

Statistical analysis revealed that the soil moisture content in the 0–20 cm layer was driven by a complex interplay of the three amendments. As shown in Table 5 and Figure 3, the moisture levels during the seedling, branching, and initial flowering stages of *Melilotus officinalis* were significantly affected not only by the primary effects of SAP, biochar, and humic acid but also by their significant pairwise and three-way interactions (*p* < 0.01). This indicates that the water-retention capacity of the soil is a synergistic result of physical structure improvement and chemical water-binding. During the seedling stage, the highest soil moisture content under single-factor conditions was observed in treatments A2, B1, and C3. For the SAP × biochar interaction, A1B1, A2B2, and A3B2 exhibited significantly higher moisture content compared with the other combinations. Under the SAP × humic acid interaction, A1C1, A2C2, and A3C3 were optimal; for the biochar × humic acid interaction, B1C3, B2C2, and B3C1 produced significantly higher values. Within the three-way interaction, A3B3C2 yielded the highest soil moisture content.

During the seedling stage, the moisture response was characterized by the dominance of SAP’s high swelling capacity (1200–1500 g/g). The highest moisture content under single-factor conditions was observed in treatments A2, B1, and C3. The significance of the SAP times biochar and SAP times humic acid interactions suggests that the porous structure of wheat straw biochar provided a framework that accommodated the expanding SAP hydrogels, preventing pore-clogging while maximizing water storage. Within the three-way interaction, the high-application combination A3B3C2 yielded the highest soil moisture content, reflecting a strong additive effect in the early growth phase.

As the plants reached the branching stage, the demand for water increased, and the regulatory role of the application rates became more pronounced to satisfy the higher transpiration demand. During this stage, A3 was significantly higher than A1 and A2 under the SAP single-factor effect; B2 and B3 exceeded B1; and C2 was significantly higher than C1 and C3. The persistence of optimal pairwise combinations (A1B1, A2B2, A3B2; A1C1, A2C2, A3C3; and B1C3, B2C2, B3C1) across stages underscores the stability of the amendment-soil matrix. Among the three-way interaction combinations, A3B3C3 performed best, demonstrating that higher doses of porous biochar and SAP are essential for maintaining the soil water reservoir during peak demand.

By the initial flowering stage, a critical shift in the optimal combination was observed, transitioning from high-dose dependence to a balanced ecological stoichiometry. During this stage, treatments A3, B3, and C1 exhibited significantly higher soil moisture content under single-factor conditions. Crucially, the three-way interaction peaked at A2B2C2, which produced the highest moisture content. This shift suggests that moderate application rates of the three factors create a more sustainable soil-water-plant equilibrium, as excessive SAP (A3) or biochar (B3) in the late stage might lead to an imbalance in soil aeration or nutrient mobility, whereas the A2B2C2 combination optimizes the coupling of water retention and availability.

#### 2.2.2. Effects of Soil Amendments on Soil pH

**Figure 4:**
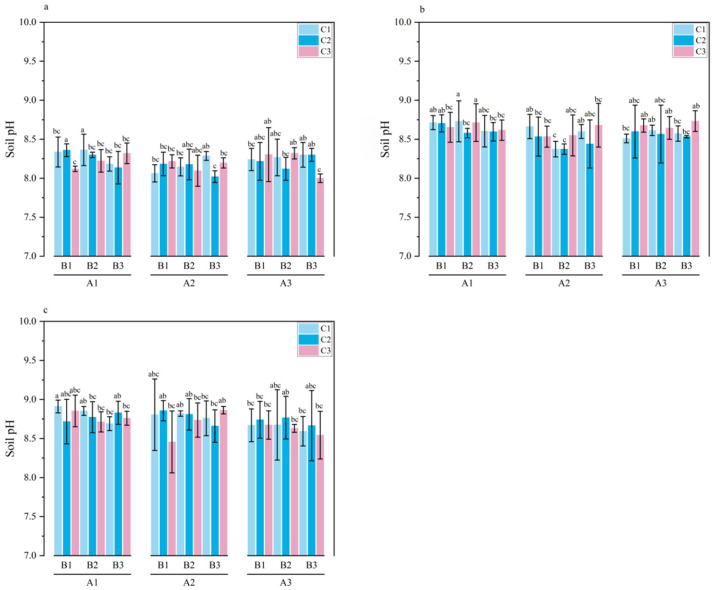
pH in 0–20 cm soil layer during the growth period of *Melilotus officinalis.* Note: Lowercase letters indicate significant differences among treatments (*p* < 0.05). Panels (**a**–**c**) represent the seedling stage, branching stage, and initial flowering stage of *Melilotus officinalis*, respectively.

The dynamic changes in soil pH within the 0–20 cm layer are summarized in Table 6 and Figure 4. The amendments demonstrated a sophisticated buffering capacity, neutralizing soil alkalinity through the synergistic interplay of chemical neutralization and moisture-mediated dilution.

During the seedling stage, the soil pH was significantly affected by the primary effect of SAP and the three-way interaction (*p* < 0.05). Under single-factor conditions, treatment A1 exhibited significantly higher pH than A2 and A3. The significant reduction in pH in the A3B3C3 treatment—the lowest among all combinations—suggests a potent initial neutralization. This can be attributed to the acidic functional groups (carboxyl and phenolic hydroxyl groups) provided by humic acid (pH 4.5–5.5), which effectively counteracted the inherent alkalinity of the wheat straw biochar and the saline–alkali soil matrix. Furthermore, the high water absorption of SAP (1200–1500 g/g) enhanced the solubility of these organic acids, creating a more uniform neutralization effect in the topsoil.

During the branching stage, the pH was influenced solely by the single-factor effect of SAP, with treatment A2 showing significantly lower pH compared with A1 and A3. This shift indicates that as the initial chemical reaction stabilized, the physical regulation of soil moisture by SAP became the dominant driver of pH stability, likely through the regulation of the soil solution’s ionic strength.

At the initial flowering stage, a complex regulatory network re-emerged, with pH significantly affected by all single factors, pairwise interactions, and the three-way interaction. The primary effects of SAP and humic acid were particularly pronounced, as the acidic nature of humic acid and the moisture-holding capacity of SAP worked in tandem to suppress resalinization-induced alkalization. Under single-factor conditions, A3 and C2/C3 treatments maintained the lowest pH levels.

The significant pairwise interactions (SAP × biochar, SAP × humic acid, and biochar × humic acid) reflect a critical ecological trade-off. For instance, in the biochar × humic acid interaction, C3 under B1/B2 exhibited significantly lower pH, confirming that humic acid’s acidity can effectively mask the alkaline nature of wheat straw biochar when applied in sufficient ratios. For the three-factor interaction, the emergence of specific optimal combinations underscores the necessity of balanced stoichiometric ratios; while treatment A2B1C3 exhibited a significantly higher pH, the overall trend suggests that the A2B2C2 or A3B3C3 combinations offer the most robust buffering against soil alkalinity by coupling organic acid release with optimized soil hydrological conditions.

#### 2.2.3. Effects of Soil Amendments on Soil Electrical Conductivity

**Figure 5:**
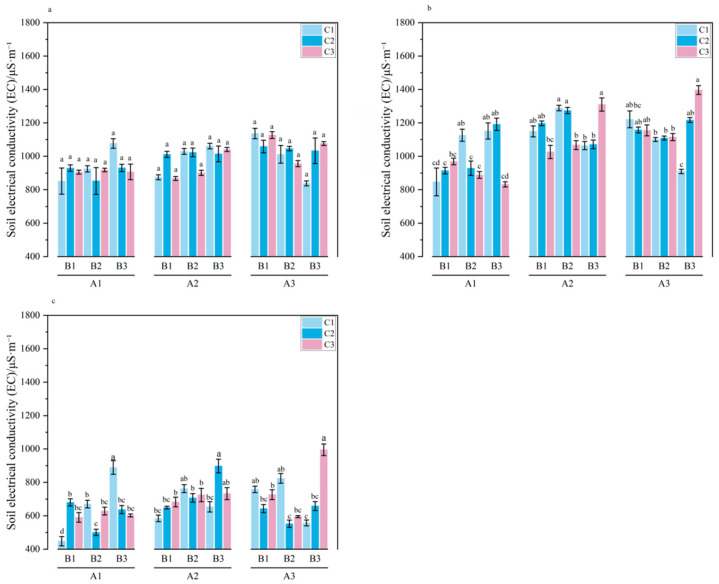
EC in 0–20 cm soil layer during the growth period of *Melilotus officinalis.* Note: Lowercase letters indicate significant differences among treatments (*p* < 0.05). Panels (**a**–**c**) represent the seedling stage, branching stage, and initial flowering stage of *Melilotus officinalis*, respectively.

The dynamic shifts in soil electrical conductivity (EC) within the 0–20 cm layer are summarized in Table 7 and Figure 5. The average EC peaked during the branching stage (1173.00 μS/cm), followed by the seedling stage (1026.78 μS/cm), and reached its minimum at the initial flowering stage (624.50 μS/cm). This “arch-shaped” trend reflects the complex kinetics of salt mobilization and leaching; the initial rise during the branching stage was likely driven by the increased solubility of salts as root activity and soil moisture peaked, while the subsequent sharp decline at the flowering stage demonstrates the cumulative leaching efficiency facilitated by the amendments (*p* < 0.01).

During the seedling stage, while the interactions between SAP, biochar, and humic acid did not yield statistically significant differences, the soil system remained in a state of hydro-chemical equilibration. Preliminary observations across single-factor levels (A1 < A2/A3; B2 < B1/B3) suggested that higher initial doses of SAP and biochar began to alter the soil’s osmotic potential, effectively preparing the profile for subsequent salt displacement.

At the branching stage, the regulatory dominance of SAP became evident. EC was significantly influenced by the primary effect of SAP and the three-way interaction (*p* < 0.05). Under single-factor conditions, A1 produced higher EC than A2 and A3. The lower EC observed in SAP-augmented treatments is directly linked to the polymer’s exceptional water absorption capacity (1200–1500 g/g), which provided sufficient solvent volume to dilute saline ions and facilitate their downward migration through the soil matrix. Within the three-way interaction, the A1B3C3 combination resulted in the highest EC, indicating that in the absence of sufficient SAP to drive leaching, the addition of high-dose wheat straw biochar may temporarily increase soil ion concentration due to its inherent ash content (12–18%).

By the initial flowering stage, the salt-leaching effect reached its maximum effectiveness, characterized by a significant dose–response relationship with SAP. The EC reduction was most pronounced in treatment A3, which exhibited a decrease of 328.68 μS/cm compared to the previous stage. The significant effects of all three amendments and their multi-way interactions at this stage underscore a robust synergistic desalinization mechanism.

These pairwise and three-way interactions reveal that the root-zone salt balance is governed by coupled physical and chemical pathways. For example, the optimal EC reduction in combinations such as A3B2 and A1B1C1 highlights a clear functional division: while SAP provides the hydraulic driving force for leaching through its swelling–desorption cycles, the wheat straw biochar provides the essential porous pathways (porosity 70–80%) for ion transport. Simultaneously, humic acid mitigated salt stress through ion exchange and chemical buffering. The superior performance of the A1B1C1 combination at this late stage suggests that a balanced, low-dose integration of these three materials can achieve sustainable salinity control, effectively managing the trade-off between amendment dosage and long-term soil remediation efficiency.

#### 2.2.4. Effects of Amendment Application on Soil Total Soluble Salt Content

**Figure 6:**
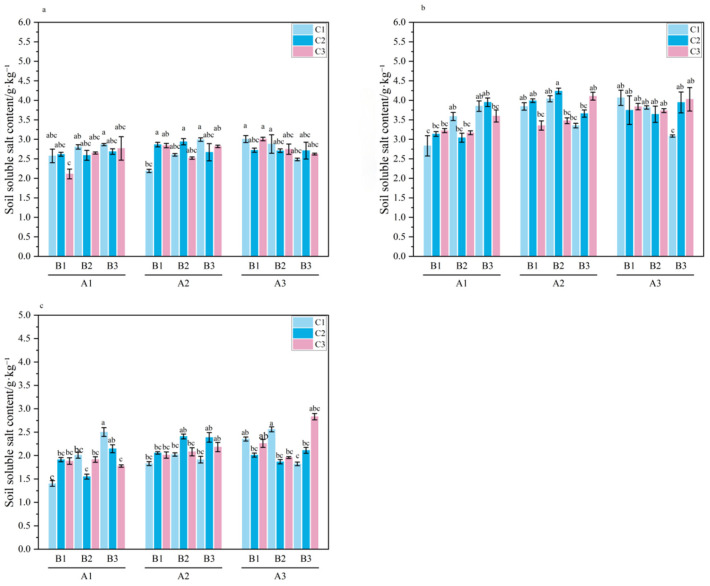
Content of soluble total salt (TSS) in 0–20 cm soil during the important growth period of *Melilotus officinalis.* Note: Lowercase letters indicate significant differences among treatments (*p* < 0.05). Panels (**a**–**c**) represent the seedling stage, branching stage, and initial flowering stage of *Melilotus officinalis*, respectively.

As shown in Table 8 and Figure 6, the total soluble salt content in the 0–20 cm soil layer exhibited a characteristic “rise-then-fall” pattern throughout the growth cycle of Melilotus officinalis. This dynamic reflects the competition between evaporative capillary rise and amendment-mediated leaching. The initial salt accumulation during the seedling and branching stages can be attributed to intense surface evaporation in the arid climate, which transported salts from deeper layers to the surface. Conversely, the reduction at the initial flowering stage indicates the combined efficacy of plant canopy shading, which suppressed evaporation, and the enhanced salt-leaching capacity facilitated by the optimized soil structure.

At the seedling stage, soil soluble salts were significantly influenced by the primary effects of biochar and humic acid, as well as the SAP × biochar and three-way interactions (*p* < 0.05). Under single-factor conditions, B1 was lower than B2 and B3. The significance of the SAP × biochar interaction (specifically in A1B1 and A2B2) suggests a structural synergy: the wheat straw biochar’s porosity (70–80%) likely provided a skeleton that prevented the hyper-expansive SAP (1200–1500 g/g) from completely sealing soil pores, thereby maintaining a balanced salt-water flux. Under the three-way interaction, A2B2C2 presented the lowest salt content, demonstrating that a balanced stoichiometric integration of these materials provides a superior buffering effect against early-stage salt accumulation compared to high-dose individual applications.

During the branching stage, total soluble salts were primarily influenced by SAP. Although A1 appeared lower than A2 and A3, this was a transient concentration effect caused by the high water-holding capacity of SAP. The polymer sequestered more moisture in the topsoil (as shown in Figure 3), temporarily concentrating soluble ions within the hydrogel matrix before subsequent leaching occurred.

At the initial flowering stage, the regulatory effects of the amendments reached a steady state, with salt content significantly affected by SAP, the SAP × humic acid interaction, and the three-way interaction. Notably, the A2B2C2 treatment consistently exhibited the lowest salt content among the three-way interactions. This highlights a critical remediation threshold: while excessive SAP (A3) might hinder drainage due to its extreme swelling, the A2B2C2 combination optimizes the trade-off between moisture retention and salt leaching. The presence of humic acid further facilitated this process through ion exchange, displacing Na^+^ from the soil complex into the leaching solution. Overall, these results confirm that the synergistic application of SAP, biochar, and humic acid not only regulates the absolute salt concentration but also optimizes the timing of salt migration to align with the salt-tolerance thresholds of *Melilotus officinalis* during its critical growth stages.

#### 2.2.5. Effects of Amendment Application on the Growth and Development of *Melilotus officinalis*

**Figure 7:**
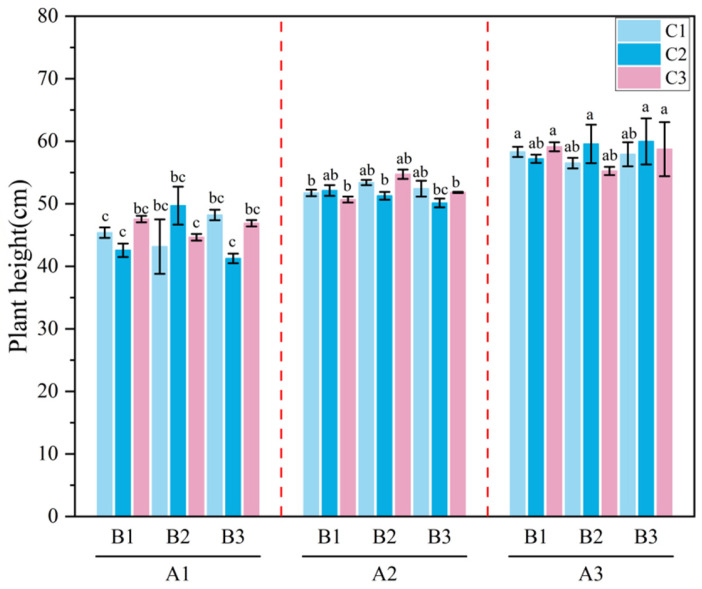
Plant Height of *Melilotus officinalis* under Different Amendment Treatments. Note: Lowercase letters indicate significant differences among treatments (*p* < 0.05).

**Figure 8:**
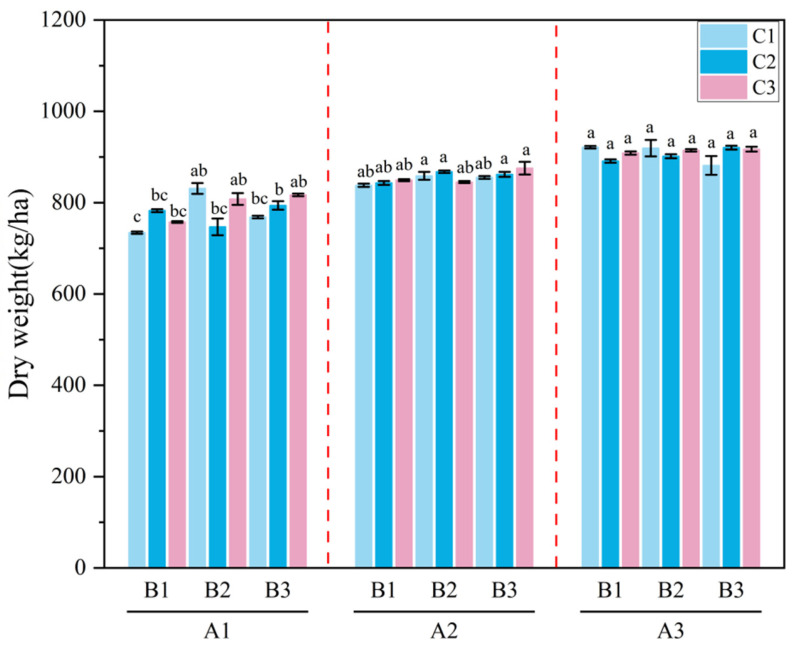
Dry Matter Yield of *Melilotus officinalis* under Different Amendment Treatments. Note: Lowercase letters indicate significant differences among treatments (*p* < 0.05). A1, A2, and A3 represent the three application levels of the superabsorbent polymer (SAP), which are 0, 30, and 60 kg/ha, respectively; B1, B2, and B3 represent the three ap-plication levels of biochar, which are 0, 15,000, and 30,000 kg/ha, respectively; and C1, C2, and C3 represent the three application levels of humic acid, which are 0, 600, and 1200 kg/ha, respectively.

As shown in Figure 7, the application of the superabsorbent polymer (SAP) significantly promoted the plant height of *Melilotus officinalis*. Specifically, the A2 and A3 treatments exhibited markedly greater height than A1, whereas under the same superabsorbent polymer level, the effects of biochar and humic acid on plant height did not differ significantly. This significant height promotion is likely attributable to the sustained water availability provided by SAP during the critical growth phases. Among all treatment combinations, A1B3C2 produced the lowest height (44.36 cm), while A3B3C3 resulted in the greatest height (60.11 cm). As illustrated in Figure 8, SAP application also significantly increased the dry biomass of *M. officinalis*, with A2 and A3 showing substantially higher dry weight than A1. Notably, while biochar and humic acid did not show significant independent effects, they exhibited a synergistic tendency with SAP; higher application rates overall led to increased biomass. Under identical SAP levels, the effects of biochar and humic acid on dry biomass were not statistically significant, although higher application rates tended to produce slightly greater dry matter accumulation. Overall, the optimal combination A2B2C2 yielded a dry biomass of 924.36 kg, representing an increase of 190.04 kg compared with A1B1C1 (734.32 kg), indicating that the applied amendments exerted a positive effect on the growth of *M. officinalis*.

## 3. Discussion

### 3.1. Effects of the Superabsorbent Polymer, Biochar, and Humic Acid on Soil Water Infiltration and Wetting Front Migration

The indoor soil column experiments demonstrated that all three soil amendments enhanced the infiltration capacity of saline–alkali soil to varying degrees [31], with the superabsorbent polymer (SAP) exerting the strongest effect, followed by biochar and humic acid [32]. Which is consistent with the findings of Abdelghafar et al. regarding the dominance of hydrogels in rapid water-holding improvement [33]. The SAP increased the water-holding capacity of the soil through swelling and water absorption [34], unlike the results of some studies in sandy soils where SAP significantly hindered deep infiltration, in this study’s saline–alkali soil, SAP acted as a “buffer reservoir” that increased the initial sorptivity (S) and stabilized the wetting front. Biochar, owing to its porous structure and high specific surface area, improved pore connectivity and increased hydraulic conductivity in the unsaturated zone, thus reducing flow resistance [35]. This aligns with Ketheeswaran et al., who reported that biochar compensates for the potential porosity reduction caused by swelling materials [36]. Humic acid improved water distribution indirectly by promoting aggregate formation [37], enhancing colloidal adsorption capacity [38], and regulating soil pH [39]. These findings indicate a functional complementarity among the three amendments in regulating soil water movement: the SAP primarily governs water storage and rapid infiltration, biochar governs pore improvement and sustained conductivity, and humic acid governs structural stabilization and uniform water distribution.

Infiltration model fitting further showed that the Kostiakov model outperformed the Philip model in simulating the temporal decline in infiltration rate [29], particularly during the steady infiltration phase, where it better captured differences in water transport among treatments [40]. This superiority is consistent with the findings of Yang et al. [41], who observed that empirical power-function models like Kostiakov are more robust in amended soils where swelling-shrinking dynamics disrupt the rigid physical assumptions of the Philip equation. Specifically, the negative values observed for the steady infiltration parameter A in the Philip model suggest that the internal swelling pressure of the SAP layers created a non-Darcian flow environment, which the standard Philip model fails to account for. When the application rates of SAP, biochar, and humic acid were 30 kg/ha, 15,000 kg/ha, and 600 kg/ha, respectively (A2B2C2), the fitting accuracy of both models reached a maximum [42], indicating that this amendment combination simultaneously optimized the initial infiltration rate and longer-term water conductivity, thereby facilitating the formation of a uniform wetting layer in saline–alkali soil.

Analysis of wetting front migration further revealed that appropriate amendment combinations substantially accelerated downward water movement while suppressing rapid surface water retention [43,44]. Although excessive amendment application (e.g., treatment T9) increased total soil water content, excessive swelling could reduce local porosity and consequently slow wetting front progression [45]. This demonstrates a clear threshold effect for amendment dosage: application rates must be sufficient to enhance initial infiltration efficiency but not so high as to induce water retention or pore obstruction.

### 3.2. Regulatory Effects of the Amendments on Soil Water–Salt Dynamics and Salt Migration

The soil water–salt distribution results showed that the combined amendments significantly promoted the downward migration of salts from the surface layer to deeper soil [46], effectively alleviating surface salt stress and improving the water–salt environment within the 0–20 cm layer [47]. This “surface desalinization and deeper accumulation” pattern is consistent with the convective-dispersive transport theory in saline soils, where amendments modify the soil solution’s mobility. Mechanistically, the superabsorbent polymer (SAP) increased the water-holding capacity of the surface soil through swelling, thereby prolonging the residence time of water in the upper layer [48]. Unlike single-material applications that may cause salt entrapment, the inclusion of biochar and humic acid in this study improved pore connectivity and macro-aggregate stability [49]. Meanwhile, biochar and humic acid improved pore structure and aggregate stability [50], enabling more uniform infiltration and consequently facilitating the downward transport of salts with percolating water [51]. This process produced a “surface desalinization and deeper accumulation” pattern of water–salt redistribution. Such coupled water–salt migration aligns with previous findings that multifunctional soil amendments enhance pore connectivity and hydraulic conditions, thereby promoting surface desalinization.

Field experiments further demonstrated that the 0–20 cm soil water content at the seedling, branching, and initial flowering stages was significantly influenced by single-factor and interactive effects of the amendments [33]. Notably, the A2B2C2 combination resulted in the highest soil water content during the seedling and branching stages [52], indicating that this specific ratio (30 kg/ha SAP, 15,000 kg/ha biochar, and 600 kg/ha humic acid) optimally aligns with the peak water demand of the crop. This provides a stable moisture reservoir that buffers the plants against osmotic stress during early development. Additionally, pH and EC analyses showed that the combined amendments regulated soil acidity–alkalinity and reduced surface salt stress [53], contributing to a sustained improvement in the soil water–salt environment.

It is noteworthy that excessive application of humic acid or the SAP (e.g., A3 or C3) did not further increase soil water content or infiltration rate [54]; rather, in some treatments, the wetting front migration was slightly reduced [55]. This “diminishing returns” effect can be attributed to the physical limitations of the soil matrix: high humic acid levels increase the soil’s dynamic viscosity, while excessive SAP swelling leads to “pore-occupying” behavior that reduces the effective cross-sectional area available for water flow. As a result, water becomes localized and “immobile,” hindering deep leaching. Therefore, when ameliorating saline–alkali soils, amendment application rates should be carefully moderated to achieve synergistic optimization rather than simple maximization.

### 3.3. Effects of the Combined Amendments on the Growth and Development of Melilotus officinalis

The improvement of the soil water–salt environment exerted a direct influence on the growth and development of *Melilotus officinalis* [56]. Field measurements showed that the combined amendment treatments significantly increased plant height and dry biomass [57], with the A2B2C2 combination (30 kg/ha SAP, 15,000 kg/ha biochar, and 600 kg/ha humic acid) performing best during both the branching and initial flowering stages [58]. This enhancement reflects the successful transition from soil hydraulic optimization to physiological promotion. Mechanistically, the increased soil water content and more uniform wetting front migration provided a continuous water supply, effectively alleviating moisture stress. Simultaneously, the reduction in surface salinity and the leaching of salts toward deeper layers decreased the osmotic inhibition of root water uptake, thereby lowering the energy expenditure required for osmoregulation and improving nutrient acquisition [59]. Meanwhile, the reduction in surface salinity and the mitigated accumulation of deeper-layer salts alleviated root-zone salt stress, decreased osmotic inhibition of root water uptake, and improved nutrient acquisition. In addition, biochar and humic acid improved soil pore structure and aggregate stability, enhanced aeration and root growth space, and created a more favorable environment for microbial activity, thereby indirectly promoting nutrient availability. Single-factor and interaction analyses indicated that the SAP had pronounced effects during the early growth stage, whereas biochar and humic acid exerted more persistent influences on water–salt regulation and soil structural improvement during the middle and late stages [60]. This demonstrates that the combined application of the three amendments not only enhances early-stage infiltration and soil moisture but also continuously improves soil conditions throughout the entire growth period, forming a coupled “water–salt–structure–plant” effect that ultimately enhances crop productivity in saline–alkali soils.

### 3.4. Synergistic Mechanisms of the Combined Amendments

Based on the results of both laboratory soil-column experiments and field trials, the combined application of the three amendments exhibits a clear synergistic effect:

(1) Synergy in pore improvement and water storage: The SAP increases pore water retention, biochar enhances pore connectivity, and humic acid stabilizes aggregate structure; together, these amendments optimize the soil hydraulic environment [61].

(2) Synergy in salt transport and pH regulation: Downward infiltration promotes the movement of salts from the surface to deeper layers, while humic acid and biochar buffer soil alkalinity and reduce soluble salt concentrations in the upper soil layer, enabling simultaneous desalinization and alkalinity mitigation.

(3) Plant physiological response: The improved water–salt environment promotes root development, enhances nutrient uptake, and improves aboveground growth parameters, ultimately contributing to increased yield [62].

Overall, the combined application of SAP, biochar, and humic acid can significantly improve the water–salt dynamics of saline–alkali soils, provide a favorable water and salt environment for plant growth, and thereby promote crop development. These findings offer both theoretical support and practical guidance for the amelioration of saline–alkali soils.

## 4. Materials and Methods

### 4.1. Laboratory Soil Column Experiment

#### 4.1.1. Experimental Materials

The soil used for the indoor soil column experiment was collected from saline–alkali land in the Wuding River Basin of Yulin, Shaanxi Province. A Z-shaped sampling strategy was employed, and soil samples were taken from a depth of 0–20 cm. After collection, the samples were manually cleaned to remove impurities, air-dried under dark conditions, and then crushed and passed through a 2 mm sieve for subsequent use. The physical and chemical properties of the initial soil are summarized as follows: the soil texture was identified as sandy loam with a bulk density of 1.39 g/cm^3^. The initial volumetric water content was 0.01 cm^3^/cm^3^. Regarding chemical properties, the soil exhibited a pH of 9.00 and an electrical conductivity (EC) of 864.45 μS/cm. The total soluble salt content was 2.70 g/kg. The soil organic carbon and total nitrogen contents were 2.89 g/kg and 0.27 g/kg, respectively, the total phosphorus content was 0.65 g/kg, the available potassium content was 0.12 g/kg. Details are provided in Table 9.

The superabsorbent polymer (SAP) used in the experiment was supplied by Shengli Oilfield Chang’an Holding Group Co., Ltd.(Dongying, China), with a high water-absorption capacity ranging from 1200 to 1500 times its own weight in distilled water. The biochar was provided by Shaanxi Yixin Bioenergy Technology Development Co., Ltd. (Yangling, China), produced from wheat straw at a pyrolysis temperature of 450 °C. The humic acid was purchased from Shanxi Hengxing Technology Co., Ltd. (Taiyuan, China), with a purity of 75%. The physicochemical properties of SAP, biochar, and humic acid are shown in Table 10.

#### 4.1.2. Experimental Apparatus and Design

**Figure 9:**
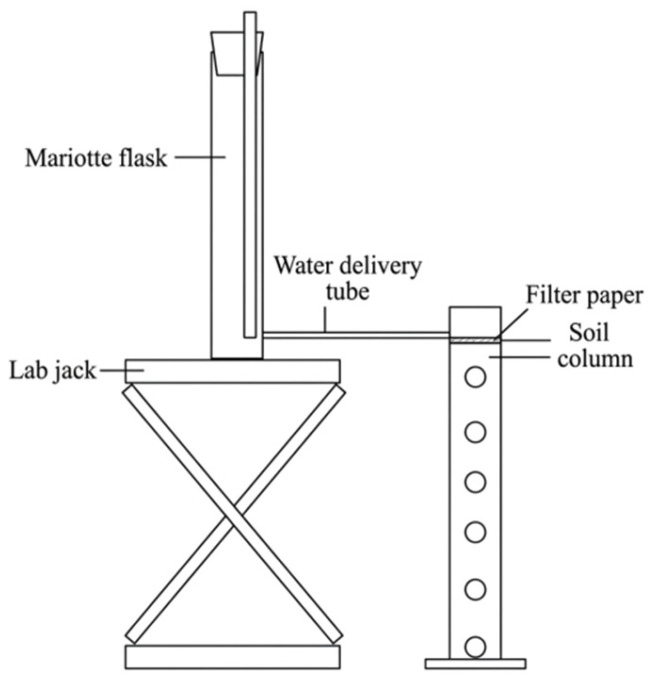
Schematic diagram of soil column infiltration device.

The infiltration experiment was conducted in a temperature-controlled laboratory maintained at 25 ± 1 °C to minimize the influence of temperature-induced viscosity changes on soil water movement. The soil column assembly consisted of cylindrical transparent acrylic tubes (inner diameter = 10 cm, height = 60 cm) [63]. The water supply system consisted of a Mariotte bottle with an inner diameter of 10 cm and a height of 40 cm. The overall setup of the experimental apparatus is shown in Figure 9. Prior to soil packing, a layer of 100-mesh nylon screen and a filter paper (diameter = 10 cm) were placed at the bottom of each column to prevent soil particle loss [64]. Additionally, a thin layer of petroleum jelly was applied to the inner walls of the columns to minimize lateral flow and edge effects during infiltration [65,66]. Soil was packed into the columns in 10 cm increments, with each layer carefully roughened and compacted to ensure uniform density. The soil was packed into the columns in 5 cm increments to reach a total height of 50 cm, targeting a consistent dry bulk density of 1.39 g/cm^3^. The required mass of soil for each layer was calculated based on the tube volume and the target density, then accurately weighed and compacted to the pre-marked lines.

Superabsorbent polymer (SAP, factor A), biochar (factor B), and humic acid (factor C) were used as the experimental amendments, each applied at three different levels. The application rates for SAP (A) were 0, 30, and 60 kg/ha, denoted as A1, A2, and A3. These rates were precisely scaled to the soil column surface area (78.54 cm^2^), corresponding to individual doses of 0 g, 0.0236 g, and 0.0471 g per column, respectively; for biochar (B), 0, 15,000, and 30,000 kg/ha, denoted as B1, B2, and B3. When converted for the laboratory setup, these amounted to 0 g, 11.7810 g, and 23.5620 g per column; and for humic acid (C), 0, 600, and 1200 kg/ha, denoted as C1, C2, and C3. Which translated to mass additions of 0 g, 0.4712 g, and 0.9425 g per column, respectively. A total of nine treatment combinations were arranged according to an orthogonal experimental design, with three replicates per treatment. The specific treatment scheme is presented in Table 11. For each treatment, the corresponding amendments were thoroughly mixed with soil and packed into the 0–20 cm layer of the columns, while the 20–50 cm layer was filled with untreated soil.

The infiltration experiment was conducted using a one-dimensional constant-head vertical ponding method [67,68]. Distilled water was used as the infiltration source, and the ponding depth was maintained at 3 cm [69]. Prior to the start of the experiment, water was added to each soil column according to its inner diameter and the initial water head to maintain the designated ponding depth, with water supplied continuously via a Mariotte bottle. Infiltration measurements were recorded according to a “dense-to-sparse” schedule: every 0.5 min for the first 3 min, every 1 min from 3 to 10 min, every 2 min from 10 to 20 min, every 4 min from 20 to 60 min, every 5 min from 60 to 100 min, every 10 min from 100 to 150 min, and every 30 min thereafter. During the experiment, the downward movement of the wetting front and changes in the water level of the Mariotte bottle were monitored using the graduated markings on the column and bottle, from which cumulative infiltration was calculated. Infiltration recording was terminated when the wetting front reached the bottom of the soil column; the water supply was then stopped, and the column was sealed. After the infiltration test, soil moisture content and soluble salt concentration were determined for each layer. Gravimetric soil water content was measured by oven-drying at 105 °C [70,71]. The dried soil samples were ground and subjected to a 1:5 soil-to-water mass ratio extraction [72,73], and the soluble salt content was determined using the residue drying method.

The measured infiltration data were fitted using the Philip theoretical infiltration model and the Kostiakov empirical infiltration equation [41,74]. The Philip infiltration model is expressed as follows:

$$I=St^{0.5}+At$$

In the equation, *I* denotes the cumulative infiltration amount, representing the total volume of water infiltrated from the onset of infiltration to time t, measured in centimeters (cm). *S* refers to the sorptivity, defined as the difference between the cumulative infiltration at the end of the first unit time interval and the steady infiltration rate. Numerically, it equals the cumulative infiltration at one minute after infiltration begins minus the steady infiltration rate, with units of cm·min^0.5^. **t** is the infiltration time, expressed in minutes (min). *A* represents the steady infiltration rate, indicating the infiltration velocity under a unit hydraulic gradient in saturated soil, or the infiltration rate when unsaturated soil reaches a relatively stable infiltration phase, measured in centimeters per minute (cm/min).

Kostiakov infiltration model

$$I=Kt^{\alpha }$$

*I* represents the cumulative infiltration amount, referring to the total infiltration from the onset of infiltration to time t, expressed in centimeters (cm). K is the infiltration coefficient, with units of cm/min. *α* denotes the infiltration exponent. *t* is the infiltration time, measured in minutes (min).

### 4.2. Field Experiment

#### 4.2.1. Field Experimental Design

**Figure 10:**
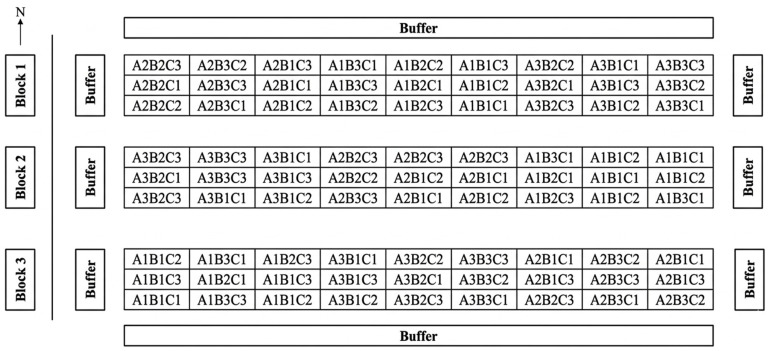
Schematic diagram of field planting of *Melilotus officinalis.*

To validate the laboratory findings under natural conditions, a multi-year fixed-site field experiment was conducted from August 2024 to June 2025 at the experimental station in Yulin, Shaanxi Province. The basic properties of the experimental plots were consistent with those of the soil used in the laboratory tests. A split–split plot design was employed, with *Melilotus officinalis* selected as the test crop and superabsorbent polymer (A), biochar (B), and humic acid (C) used as soil amendments. The application rates for superabsorbent polymer (SAP), biochar, and humic acid were selected based on a combination of preliminary experimental results, existing literature on saline–alkali soil remediation, and practical agronomic considerations. Three application rates of the superabsorbent polymer were assigned to the main plots: 0, 30, and 60 kg/ha, designated as A1, A2, and A3, respectively. Three biochar application rates were assigned to the split plots: 0, 15,000, and 30,000 kg/ha, designated as B1, B2, and B3. Three humic acid application rates were assigned to the split–split plots: 0, 600, and 1200 kg/ha, designated as C1, C2, and C3. In total, 27 treatments were established, each with three replicates, with A1B1C1 serving as the control (CK). Each plot covered an area of 4 m^2^ (2 m × 2 m), and the specific planting layout is shown in Figure 10. Row spacing for drill seeding was 25 cm, with a seeding rate of 30 kg/hm^2^. Sowing was conducted in early August 2024. Biochar, humic acid, and the superabsorbent were uniformly broadcasted onto the surface and incorporated into the top 0–20 cm of the soil matrix using a rotary tiller to ensure a homogeneous mixture. Field management, including weeding and pest control, was conducted uniformly across all plots according to local agronomic practices. Weed control was performed manually on 25 August and 20 September. Pest management (primarily for aphids) was executed using 10% imidacloprid on 5 September. No chemical growth regulators were used.

#### 4.2.2. Field Measurement Parameters

Field soil sampling: During the seedling, branching, and initial flowering stages of *Melilotus officinalis*, soil samples from the 0–20 cm layer were collected using the five-point sampling method to determine soil moisture content, pH, soluble salt ion concentrations, and electrical conductivity. Plant growth parameters were measured at the initial flowering stage, including plant height and shoot dry weight of *M. officinalis.* The specific methods are as follows:

(1) Soil moisture content: Soil water content was determined using the oven-drying method. Fresh soil samples were dried in an oven at 105 ± 2 °C to a constant weight, and gravimetric water content was calculated accordingly.

(2) Soil bulk density: Soil bulk density was measured in situ using the cutting-ring (core) method. Values were obtained based on the known volume of the ring sampler and the oven-dried soil mass.

(3) Soil pH measurement: Soil pH was determined using a soil-to-deionized water suspension at a ratio of 2.5:1. Deionized water was boiled to remove dissolved CO_2_ before use. After thorough shaking and equilibration, pH was measured using a Mettler FiveEasy Plus FE28 pH meter(Mettler Toledo, Zurich, Switzerland).

(4) Electrical conductivity (EC): Soil EC was measured using a 5:1 (water-to-soil) extract prepared with deionized water. The supernatant was analyzed using a conductivity meter, and EC was used as an indicator of total soluble salt content in the soil.

(5) Plant growth parameters: Plant sampling was conducted at the initial flowering stage of *Melilotus officinalis*. In each experimental plot, three representative sampling quadrats (70 cm × 100 cm) with uniform growth conditions were randomly selected, and all aboveground biomass within each quadrat was harvested. Fresh weight was recorded immediately after harvest. Approximately 1.5 kg of representative fresh plant material was randomly subsampled from each quadrat and air-dried in a ventilated environment. Subsequently, plant samples were separated into aboveground and belowground parts, and surface contaminants were removed by rinsing with deionized water. The samples were first blanched at 105 °C to inactivate enzymatic activity and then oven-dried at 65 °C for 72 h until constant weight was achieved. After drying, samples were cooled in a desiccator for 0.5 h to reach room temperature. Dry biomass was determined using an analytical balance (precision: 0.0001 g), and all data were recorded in a database for further analysis.

#### 4.2.3. Data Processing and Statistical Analysis

For scientific quantification of the overall contribution of different amendment treatments to saline–alkali soil reclamation, a fuzzy membership function analysis was introduced to construct a multi-dimensional evaluation model. This approach transforms individual physicochemical indicators into membership values ranging from 0 to 1, thereby enabling the normalization of variables with different dimensions.

For indicators that contribute positively to soil fertility, an ascending membership function was applied for transformation, and the calculation was conducted according to Equation (1). For indicators that negatively affect soil quality as their values increase, a descending membership function was used for transformation, with calculations performed according to Equation (2).

(1) R(Xij)_positive_ = (Xij − Xjmin)/(Xjmax − Xjmin)

(2) R(Xij)_negative_ = 1 − [(Xij − Xjmin)/(Xjmax − Xjmin)]

In the equation, X_ij_ represents the observed value of the j-th indicator under the i-th treatment, while X_jmin_ and X_jmax_ denote the minimum and maximum values of the j-th indicator, respectively.

Data visualization was performed using Origin 2021 software, while statistical analyses were conducted using SPSS 21.0 (IBM Corp., Armonk, NY, USA). One-way analysis of variance (One-way ANOVA) was applied to test significant differences among treatments.

## 5. Conclusions

The combined application of superabsorbent polymer (SAP), biochar, and humic acid significantly optimizes the water–salt environment and crop productivity in saline–alkali soils. SAP primarily drives rapid infiltration and water retention, while biochar and humic acid provide structural stability and alkalinity buffering, facilitating a “surface desalinization and deeper accumulation” pattern. However, a dosage threshold exists; excessive application leads to pore-clogging and localized water retention, hindering salt leaching. This study identifies the A2B2C2 combination (30 kg/ha SAP, 15,000 kg/ha biochar, and 600 kg/ha humic acid) as the optimal ratio, which consistently achieves the best balance between hydraulic conductivity, soil structure, and the growth of *Melilotus officinalis* across both laboratory simulations and field trials. These findings establish a synergistic “water–salt–structure–plant” regulatory framework, providing a scientific and practical basis for the efficient remediation of saline–alkali landscapes.

## Figures and Tables

**Table 1:** L_9_(3^3^) orthogonal design.

	Level			Cumulative Infiltration/cm			100 min Wet Front Migration/cm			300 min Wet Front Migration/cm		
Treatment	A	B	C									
T1	1	1	1	18.95	19.23	20.06	17.00	23.00	24.70	32.70	40.00	30.00
T2	1	2	3	19.82	20.37	20.53	19.80	21.80	22.40	35.30	39.00	38.00
T3	1	3	2	19.25	19.95	20.26	18.00	19.30	16.70	34.30	35.20	34.00
T4	2	1	3	19.63	20.20	20.45	18.00	23.40	18.90	36.30	39.50	39.60
T5	2	2	2	19.32	20.53	20.32	20.30	18.60	24.10	35.00	29.90	40.00
T6	2	3	1	19.36	20.10	20.54	19.50	16.80	18.40	34.00	31.60	31.20
T7	3	1	2	19.12	19.20	20.02	19.50	19.50	18.30	32.50	32.40	31.80
T8	3	2	1	18.67	19.35	19.91	19.40	27.50	23.20	33.50	40.00	37.70
T9	3	3	3	18.83	19.21	19.26	16.80	16.90	16.90	30.20	26.90	26.40

Note: A represents the superabsorbent polymer (SAP), B represents biochar, and C represents humic acid. The numbers 1, 2, and 3 indicate different application rates. The three application levels of the superabsorbent polymer (SAP) were 0, 30, and 60 kg/ha; the three biochar application levels were 0, 15,000, and 30,000 kg/ha; and the three humic acid application levels were 0, 600, and 1200 kg/ha.

**Table 2:** Analysis of cumulative infiltration range for each treatment.

Item	Level	Super Absorbent Polymer (A)	Biochar B	Humic Acid C
Sum of cumulative infiltration	K1	178.42	176.86	176.41
	K2	180.45	178.82	177.97
	K3	173.57	176.76	178.30
Average of cumulative infiltration	k1	19.82	19.65	19.60
	k2	20.05	19.86	19.77
	k3	19.28	19.64	19.81
Rank		0.77	0.22	0.21

**Table 3:** Analysis of the wetting‑front range for each treatment.

Item	Level	Super Absorbent Poly A	Biochar B	Humic Acid C
Wetting front migration distance at 100 min/cm	K1	182.70	189.51	182.7
	K2	178.00	174.30	177.99
	K3	159.30	174.90	178.02
	k1	20.30	21.06	20.3
	k2	19.70	19.37	19.78
	k3	17.70	19.43	19.78
	R	2.60	1.69	0.52
Wetting front migration distance at 300 min/cm	K1	314.79	318.48	310.71
	K2	328.41	317.13	305.10
	K3	283.80	291.39	311.19
	k1	34.98	35.39	34.52
	k2	36.49	35.24	33.90
	k3	31.53	32.38	34.58
	R	4.96	3.01	0.68

**Table 4:** Estimation of parameter values for dual infiltration models.

	Philip Model			Kostiakov Model		
Treatment	S	A	*R* ^2^	K	a	*R* ^2^
T1	0.914356	−0.0053	0.994798	0.976735	0.445335	0.99713
T2	1.147915	−0.01713	0.993664	1.459238	0.40886	0.99759
T3	0.769694	−0.00601	0.99597	0.950899	0.434932	0.998508
T4	0.98835	−0.00533	0.99183	1.138324	0.456708	0.994679
T5	0.840898	−0.00293	0.998437	0.906001	0.475591	0.999003
T6	0.760917	−0.00196	0.995521	0.83831	0.473828	0.996736
T7	0.761461	0.000329	0.998847	0.774586	0.497735	0.998847
T8	0.922342	−0.00336	0.987493	1.060512	0.461803	0.990406
T9	0.671856	0.005515	0.998588	0.621891	0.53604	0.997666

**Table 5:** Effects of Super Absorbent Polymer, Biochar, and Humic Acid on Soil Moisture Content (0–20 cm) during Key Growth Stages of *Melilotus officinalis.*

Treatments	Soil Moisture Content (%)		
	Seedling Stage	Branching Stage	Initial Flowering Stage
A1	22.82 ± 0.61	20.92 ± 0.81	22.65 ± 0.48
A2	23.24 ± 2.33	20.56 ± 0.84	22.58 ± 0.93
A3	23.20 ± 1.59	21.64 ± 1.94	22.76 ± 2.01
B1	23.21 ± 1.72	20.52 ± 0.53	22.62 ± 0.89
B2	22.94 ± 1.35	21.37 ± 1.78	22.46 ± 1.45
B3	23.04 ± 2.06	21.02 ± 0.76	23.00 ± 0.99
C1	23.14 ± 1.89	20.92 ± 0.91	23.02 ± 1.14
C2	22.55 ± 0.91	21.12 ± 1.84	22.20 ± 1.23
C3	23.52 ± 1.90	20.86 ± 0.76	22.74 ± 0.97
ANOVA			
A	**	**	**
B	**	**	**
C	**	**	**
A × B	**	**	**
A × C	**	**	**
B × C	**	**	**
A × B × C	**	**	**

Note: ** means significant difference *p* < 0.01. A1, A2, and A3 represent the three application levels of the superabsorbent polymer (SAP), which are 0, 30, and 60 kg/ha, respectively; B1, B2, and B3 represent the three ap-plication levels of biochar, which are 0, 15,000, and 30,000 kg/ha, respectively; and C1, C2, and C3 represent the three application levels of humic acid, which are 0, 600, and 1200 kg/ha, respectively.

**Table 6:** Effects of Super Absorbent Polymer, Biochar, and Humic Acid on Soil pH (0–20 cm) during Key Growth Stages of *Melilotus officinalis.*

Treatments	Soil pH		
	Seedling Stage	Branching Stage	Initial Flowering Stage
A1	8.26 ± 0.10	8.66 ± 0.06	8.79 ± 0.08
A2	8.15 ± 0.08	8.53 ± 0.11	8.75 ± 0.97
A3	8.23 ± 0.11	8.61 ± 0.07	8.66 ± 0.27
B1	8.23 ± 0.10	8.62 ± 0.08	8.74 ± 0.96
B2	8.22 ± 0.09	8.57 ± 0.13	8.70 ± 1.00
B3	8.19 ± 0.12	8.60 ± 0.08	8.75 ± 0.98
C1	8.24 ± 0.10	8.60 ± 0.11	8.76 ± 0.30
C2	8.20 ± 0.11	8.55 ± 0.10	8.69 ± 1.00
C3	8.20 ± 0.11	8.65 ± 0.07	8.69 ± 0.13
ANOVA			
A	*	*	**
B	ns	ns	ns
C	ns	ns	**
A × B	ns	ns	**
A × C	ns	ns	**
B × C	ns	ns	**
A × B × C	*	ns	**

Note: * means significant difference *p* < 0.05, ** means significant difference *p* < 0.01, “ns” means no significant difference. A1, A2, and A3 represent the three application levels of the superabsorbent polymer (SAP), which are 0, 30, and 60 kg/ha, respectively; B1, B2, and B3 represent the three application levels of biochar, which are 0, 15,000, and 30,000 kg/ha, respectively; and C1, C2, and C3 represent the three application levels of humic acid, which are 0, 600, and 1200 kg/ha, respectively.

**Table 7:** Effects of Super Absorbent Polymer, Biochar, and Humic Acid on Soil Electrical Conductivity (0–20 cm) during Key Growth Stages of *Melilotus officinalis.*

Treatments	Soil Electrical Conductivity (µS/cm)		
	Seedling Stage	Branching Stage	Initial Flowering Stage
A1	921.93 ± 65.59	983.30 ± 137.00	624.50 ± 124.00
A2	980.44 ± 76.60	1173.00 ± 110.00	710.50 ± 88.30
A3	1026.78 ± 91.16	1154.00 ± 129.00	698.10 ± 144.00
B1	973.67 ± 112.53	1071.00 ± 135.00	640.20 ± 91.80
B2	962.78 ± 68.03	1100.00 ± 133.00	662.70 ± 104.00
B3	997.70 ± 85.26	1127.00 ± 180.00	736.00 ± 154.00
C1	978.44 ± 108.78	1095.00 ± 140.00	683.00 ± 140.00
C2	989.00 ± 68.97	1118.00 ± 126.00	658.40 ± 110.00
C3	966.70 ± 91.70	1084.00 ± 185.00	697.50 ± 126.00
ANOVA			
A	ns	**	**
B	ns	ns	**
C	ns	ns	**
A × B	ns	ns	**
A × C	ns	ns	**
B × C	ns	ns	**
A × B × C	ns	*	**

Note: * means significant difference *p* < 0.05, ** means significant difference *p* < 0.01, “ns” means no significant difference. A1, A2, and A3 represent the three application levels of the superabsorbent polymer (SAP), which are 0, 30, and 60 kg/ha, respectively; B1, B2, and B3 represent the three ap-plication levels of biochar, which are 0, 15,000, and 30,000 kg/ha, respectively; and C1, C2, and C3 represent the three application levels of humic acid, which are 0, 600, and 1200 kg/ha, respectively.

**Table 8:** Effects of Super Absorbent Polymer, Biochar, and Humic Acid on Soil Total Soluble Salts (0–20 cm) during Key Growth Stages of *Melilotus officinalis.*

Treatments	Soil Total Soluble Salt (g/kg)		
	Seedling Stage	Branching Stage	Initial Flowering Stage
A1	2.85 ± 0.75	3.38 ± 0.38	1.90 ± 0.32
A2	2.72 ± 0.25	3.79 ± 0.34	2.10 ± 0.20
A3	2.77 ± 0.17	3.77 ± 0.29	2.20 ± 0.34
B1	2.66 ± 0.33	3.56 ± 0.43	2.04 ± 0.27
B2	2.62 ± 0.14	3.54 ± 0.38	1.97 ± 0.30
B3	2.96 ± 0.70	3.73 ± 0.34	2.19 ± 0.34
C1	2.71 ± 0.27	3.61 ± 0.43	2.05 ± 0.37
C2	2.70 ± 0.11	3.61 ± 0.40	2.03 ± 0.26
C3	2.90 ± 0.75	3.62 ± 0.34	2.10 ± 0.31
ANOVA			
A	ns	*	**
B	*	ns	ns
C	ns	ns	ns
A × B	**	ns	ns
A × C	ns	ns	*
B × C	*	ns	ns
A × B × C	**	ns	**

Note: * means significant difference *p* < 0.05, ** means significant difference *p* < 0.01, “ns” means no significant difference. A1, A2, and A3 represent the three application levels of the superabsorbent polymer (SAP), which are 0, 30, and 60 kg/ha, respectively; B1, B2, and B3 represent the three ap-plication levels of biochar, which are 0, 15,000, and 30,000 kg/ha, respectively; and C1, C2, and C3 represent the three application levels of humic acid, which are 0, 600, and 1200 kg/ha, respectively.

**Table 9:** The soil basic physical properties.

Soil Type	Soil Buik Density/g/cm^3^	Soil Initial Water Content/cm^3^/cm^3^	EC/μS/cm	pH	Total Soluble Salt/g/kg	Soil Organic Carbon/g/kg	Soil Total Nitrogen/g/kg	Soil Total Nitrogen Conten/g/kg
Sandy loam	1.39	0.01	864.45	9.00	2.70	2.89	0.27	0.65

**Table 10:** Physicochemical properties of the superabsorbent polymer (SAP), biochar, and humic acid used in the study.

Material	Property	Value
SAP	Main Chemical Component	Sodium Polyacrylate/Polyacrylamide
	Particle size (mm)	0.5–2.0
	Water absorption (deionized water, g/g)	1200–1500
	Water absorption (0.9% NaCl solution, g/g)	80–120
	Bulk density (g/cm^3^)	0.7
Biochar	Feedstock	Wheat straw
	Pyrolysis temperature (°C)	450
	Specific surface area (BET, m^2^/g)	200
	Porosity (%)	70
	pH (1:10 water)	9.5
	Ash content (%)	15
Humic Acid	Humic acid content (%)	≥75%
	Water solubility	Fully soluble
	pH (1:100 water)	4.5
	Carboxyl group content (mmol/g)	4
	Phenolic hydroxyl group content (mmol/g)	2.5

**Table 11:** Factor and level table.

Treatment		Factor	
	Super Absorbent Polymer (A)/kg/ha	Biochar (B)/kg/ha	Humic Acid (C)/kg/ha
T1 (A1B1C1)	0	0	0
T2 (A1B2C3)	0	15,000	1200
T3 (A1B3C2)	0	30,000	600
T4 (A2B1C3)	30	0	1200
T5 (A2B2C2)	30	15,000	600
T6 (A2B3C1)	30	30,000	0
T7 (A3B1C2)	60	0	600
T8 (A3B2C1)	60	15,000	0
T9 (A3B3C3)	60	30,000	1200

## Data Availability

The original contributions presented in this study are included in the article. Further inquiries can be directed to the corresponding author.
